# High-dose ascorbate and arsenic trioxide selectively kill acute myeloid leukemia and acute promyelocytic leukemia blasts *in vitro*

**DOI:** 10.18632/oncotarget.15925

**Published:** 2017-03-06

**Authors:** Nélida I. Noguera, Elvira Pelosi, Daniela F. Angelini, Maria Liliana Piredda, Gisella Guerrera, Eleonora Piras, Luca Battistini, Lauretta Massai, Anna Berardi, Gianfranco Catalano, Laura Cicconi, Germana Castelli, Agnese D’Angiò, Luca Pasquini, Grazia Graziani, Giuseppe Fioritoni, Maria Teresa Voso, Domenico Mastrangelo, Ugo Testa, Francesco Lo-Coco

**Affiliations:** ^1^ Department of Biomedicine and Prevention, University of Rome Tor Vergata, Rome, Italy; ^2^ Santa Lucia Foundation, I.R.C.C.S. Via del Fosso di Fiorano, Rome, Italy; ^3^ Department of Hematology, Oncology and Molecular Medicine, Istituto Superiore di Sanità, Rome, Italy; ^4^ Neuroimmunology and Flow Cytometry Units, Fondazione Santa Lucia-I.R.C.C.S., Rome, Italy; ^5^ Department of Medical, Surgical and Neurological Sciences, University of Siena, Polo Scientifico San Miniato, Siena, Italy; ^6^ Pescara Cell Factory Foundation Onlus, Pescara, Italy; ^7^ Department of Systems Medicine, University of Rome Tor Vergata, Rome, Italy

**Keywords:** high-dose ascorbate, arsenic trioxide, acute promyelocytic leukemia, acute myeloid leukemia

## Abstract

The use of high-dose ascorbate (ASC) for the treatment of human cancer has been attempted several decades ago and has been recently revived by several *in vitro* and *in vivo* studies in solid tumors. We tested the cytotoxic effects of ASC, alone or in combination with arsenic trioxide (ATO) in acute myeloid leukemia (AML) and acute promyelocytic leukemia (APL). Leukemic cell lines and primary blasts from AML and APL patients were treated with graded concentrations of ASC, alone or in association with standard concentration (1 μM) of ATO. The ASC/ATO combination killed myeloid blasts, including leukemic CD34^+^ cells, while sparing CD34^+^ progenitors obtained from normal cord blood and bone marrow. Actually, approximately one-third (11/36) of primary AML cases were highly sensitive to the ASC/ATO combination. The mechanism of cell killing appeared to be related to increased oxidative stress and overproduction of ROS in a non-quantitative fashion, which resulted in induction of apoptosis. These effects were reverted by the addition of the antioxidant N-Acetyl-Cysteine (NAC). In the APL NB4 model, ASC induced direct degradation of the PML and PML/RARA proteins via caspase activation, while the transcriptional repressor DAXX was recruited in re-constituted PML nuclear bodies. Our findings encourage the design of pilot studies to explore the potential clinical benefit of ASC alone or in combination with ATO in advanced AML and APL.

## INTRODUCTION

Reactive Oxygen Species (ROS) are continuously generated in human cells, where they act as secondary signaling messengers, and are required for a number of metabolic processes [1–5]. ROS production and elimination through scavenging systems is tightly regulated to maintain redox homeostasis: an increase in ROS levels results in oxidative stress with aberrant cell signaling and damage, while a decrease in ROS leads to disruption of cell signaling [6]. Most cancer cells are characterized by ROS overproduction, oxidative challenge and unusually high antioxidant levels to balance it [7]. Normal cells, producing ROS at a lower rate, require lower amount of ROS-scavenging proteins compared to cancer cells and are therefore more resistant to exogenously increased oxidative stress. Redox modulation as cancer therapy is a still controversial but rapidly growing research field, with promising results reported in some pre-clinical studies [8–12]. Recently, Yun et al. reported that cultured human colorectal cancer cells harboring KRAS or BRAF mutations are selectively killed by high doses of vitamin C [13].

Vitamin C (ascorbic acid) is an essential micronutrient in humans, and is one of a large variety of dietary antioxidants. *In vitro* evidence suggests that ascorbate (ascorbic acid in solution) functions as antioxidant at low concentrations, but has pro-oxidant activity at high concentrations [14]. Originally thought to be protective against tumors [15–16], ascorbic acid at high concentrations (hereafter referred to as ASC) was reported by Cameron and Pauling to have therapeutic effects in patients with terminal cancer [17]. Subsequent studies using ASC given orally did not confirm these results [18–19]. However, pharmacokinetic studies on ASC indicate a self-limiting intestinal absorption, with blood concentration reaching about 100 μM after oral administration of 0.4 g [11, 20]. Parenteral administration is needed to reach the 3 to 20 mM blood concentrations necessary to obtain the pro-oxidant function required for therapeutic effect [20]. Although there is some evidence for a therapeutic efficacy of intravenous (IV) ASC, its clinical benefit has remained elusive, and based on single or few case-reports [21–27]. A number of clinical trials are currently ongoing to ascertain the pharmacodynamic properties of IV ASC and its possible role in cancer treatment.

Acute myeloid leukemia (AML) is a hematopoietic neoplasm mainly affecting elderly individuals. With the exception of some subtypes such as acute promyelocytic leukemia (APL) and the core binding factor AML, the prognosis of the disease is usually dismal with 5-year survival rates of only 5–10% in patients aged > 60 years [28–29]. By contrast, APL is nowadays curable in the vast majority of cases and is highly responsive to targeted agents including all trans retinoic acid (ATRA) and arsenic trioxide (ATO) [30]. These agents bind to the two moieties of the disease-specific oncoprotein PML/RARA. Moreover, ATO induces the formation of nuclear matrix-associated nuclear bodies (NBs) from PML/RARA multimers, which are then degraded, finally affecting the oxidative status of target cells [31].

We recently reported that ASC induces apoptosis *in vitro* in a variety of human myeloid cell lines including ATRA-resistant and ATO-resistant cell lines, while it neither exerted significant cytotoxic effects, nor impaired the differentiation potential in cord blood-derived CD34^+^ normal cells [32]. To further investigate the role of ASC in the treatment of AML, we extended our studies to explore the effects of ASC in combination with a standard concentration (1 μM) of ATO, again using normal hematopoietic CD34^+^ cells as controls. In addition to cell lines, primary blasts obtained from AML and APL patients were challenged with ASC + ATO.

## RESULTS

### Effects of ASC +/−ATO on survival of leukemic cell lines and primary blasts

**Figure 1:**
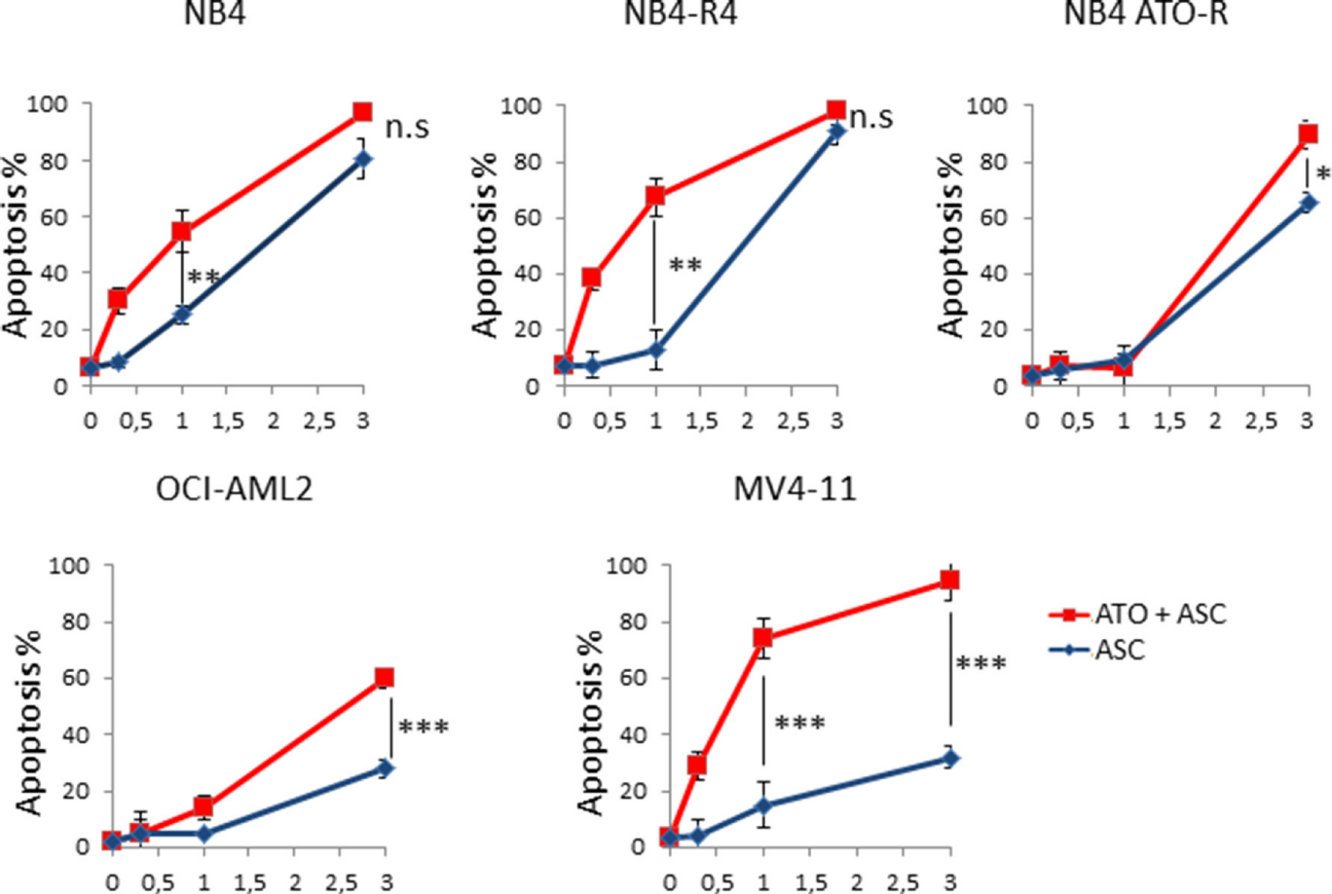
Effects of ASC and ATO on survival of leukemic cell lines Cell death induced by 1 μM ATO and increasing concentrations of ASC (0, 0.3, 1 e 3 mM) was evaluated at 48 h in NB4, NB4-R4, NB4 ATO-R, Oci-AML2, and MV4;11 leukemia cell lines by flow cytometry after Annexin V - PI staining. Values are expressed as mean ± SE of three independent experiments. Statistical analysis was performed using the Anova *t* test and Tukey's Multiple Comparison Test *p ≤ 0.01; **p ≤ 0.001; ***p ≤ 0.0001; ns: not significant.

Increasing doses of ASC were tested, starting at 300 μM and scaling up to 3 mM. Marked decrease of cell proliferation was initially detected in NB4 and NB4-R4 cells using 1 mM ASC (data not shown), whereas 3 mM ASC induced a significant increase in apoptosis not only in NB4, NB4-R4 but also in NB4 ATO-R cells. In the cell lines, less sensitive to ASC as single agent (i.e, Oci-AML2 and MV4;11) the combination of 1 μM ATO with ASC (3 mM in Oci-AML2; 1 and 3 mM in MVA;11) resulted in a remarkable increase in the percentage of apoptotic cells, as shown by Annexin V–PI staining after 48 h (Figure 1).

We calculated the Combination index analysis of ASC/ATO in NB4, NB4-ATO-R and Oci-AML2 cell lines. The cell lines were selected as models of PML/RARA+ APL and non-APL AML respectively. In NB4 cells the 50% inhibitory concentration (IC_50_) of ASC as single agent was 1.3 ± 0.3 mM, whereas the IC_50_ of ASC in combination with ATO was 0.8 ± 0.07 mM. In NB4-ATO-R cells, the IC_50_ was similar when using ASC or the ASC/ATO combination (3.77 ± 1.13 mM and 4.00 ± 1.24 mM respectively, *p* = 0.84). In Oci-AML2, the ASC IC_50_ was 4.5 ± 0.31 mM, and the addition of ATO to ASC almost halved the ASC IC_50_ (2.4 ± 0.48 mM) (mean ± SE of three independent experiments). For the evaluation of the combination index (CI) the cell lines were treated with a fixed non-toxic concentration of ATO (i.e, 0.5 μM for NB4 and NB4-ATO-R, and 1 μM for Oci-AML2) and graded concentrations of ASC. In Oci-AML2 cells the drug combination resulted in synergistic effects with CI values > 1 at all combination data points, while in NB4, effects were only slightly additive (Table 1).

**Table 1:** Additive or synergistic effects of the ATO/ASC combination on cell lines

NB4 cell line	CI	OCI-AML2 cell line	CI
μm ATO + 1.5 mM ASC	1.0 ± 0.1	1 μm ATO + 3 mM ASC	0.9 ± 0.17
0.5 μm ATO + 2 mM ASC	1.0 ± 0.1	1 μm ATO + 5 mM ASC	0.9 ± 0.12
0.5 μm ATO + 2.5 mM ASC	0.94 ± 0.04	1 μm ATO + 7 mM ASC	0.5 ± 0.27
0.5 μm ATO + 3 mM ASC	0.95 ± 0.06	1 μm ATO + 8 mM ASC	0.5 ± 0.16

CI: Combination Index.

**Figure 2:**
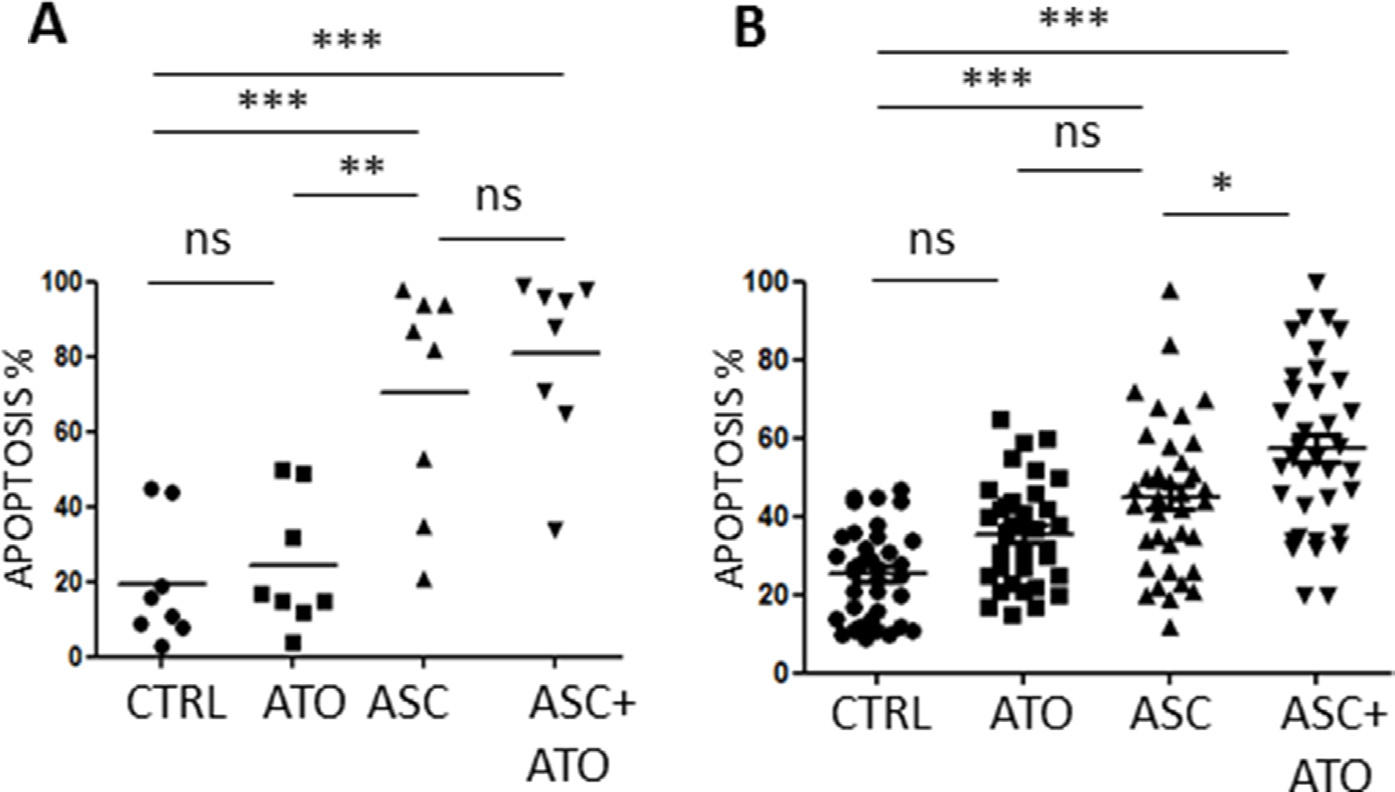
Apoptotic effects of ASC+/−ATO in primary leukemic cells Primary APL and AML cells were treated with 1 μM ATO, 3 mM ASC or 1 μM ATO + 3 mM ASC and analyzed by FACS after Annexin V-PI staining. PML/RARA positive APL samples (**A**), AML samples (**B**), Statistical analysis was performed using the Anova *t* test and Tukey's Multiple Comparison Test; *p ≤ 0.01; **p ≤ 0.001; ***p ≤ 0.0001; ns: not significant.

We then treated primary leukemic blasts using 3 mM ASC alone or in combination with 1 μM ATO, for 48 h. Primary APL blasts were efficiently killed by ASC alone (mean 70.5% ± 29.9% apoptosis), whereas AML blasts showed variable response rates (44.9 ± 19.3%, Figures 2A and 2B and Table 2). In the AML group, we could identify a group of “highly sensitive” (HS) samples in which > 60% apoptosis was induced by the ASC/ATO combination, accounting for about 30% of tested AML cases (Table 2). The main characteristics of the ASC-sensitive patient group were heterogeneous, with no predictable association between ASC sensitivity and biologic features (Table 3). No significant effects on myeloid differentiation were seen, as we did not observe significantly changes in CD11, CD14 and CD15 expression during treatment (data not shown). Efficient killing was also observed in two CML cases, and especially in the sample of CML blast crisis (Table 2).

**Table 2:** Percentages of apoptotic cells following culture of patient-derived leukemia samples in the presence of ASC and/or ATO

Patient samples (*n*)	Control (% ± SD)	1 μM ATO (% ± SD)	3 mM ASC (% ± SD)	1 μM ATO + 3 mM ASC (% ± SD)
**PML/RARA+ APL (*n* = 8)**	19.4 ± 16.3	24.3 ± 17.4	70.5 ± 29.9	80.8 ± 22.8
**AML (*n* = 26)**	25.5 ± 11.7	35.5 ± 13.1	44.9 ± 19.3	57.4 ± 21.3
**HS-AML (*n* = 11)**	26.7 ± 12.4	39.8 ± 12.8	62.6 ± 17.6	81.5 ± 10.8
**CML (*n* = 1)**	4	8	38	47
**CML-BC (*n* = 1)**	29	61	76	90

HS-AML, Highly sensitive AML. CML-BC, chronic myeloid leukemia blast crisis.

**Table 3:** Characteristics of Highly Sensitive AML samples (HS-AML)

N°	Blast (%)	*FLT3*	*NPM1*	*AML1-ETO*	*CBFbeta/MYH11*	Karyotype	Immunophenotype	WBC	AGE	ELN-RISK
1	70	NEG	NEG	NEG	NEG	49,XX,+8,+mar1,+mar2[7] 46,XX[13]	CD34/33+/64+/11b+/4+/DR+/15+/47­/13-/56+/14-	27.7 × 109		High
1	69	NEG	NEG	NEG	NEG	46,XX [20]	CD45+/33+/34+/DR+/7+/15+/117+/13+/3/8+	2440	49	Intermediate
3	80	NEG	NEG	NEG	NEG	45,X,-Y[14] 46,XX[6]	CD45+/34+/33-/DR+/117+/15-/13+/38+	6780	41	Intermediate
4	73	NEG	NEG	POS	NEG	46,XY, t(8;21)	CD45­/34+/DR+/33±/117+/15+/−13+/−56+	4700	32	Low
5	78	NEG	NEG	NEG	NEG	46,XX,del(12)(p11p13)[13]/46,XX[7]	CD34+/33+/DR±/7+/117+/15-/13+/38+	2700	32	Intermediate
6	71	NEG	POS	NEG	NEG	46,XX[20]	CD34­/33+/15±/117+/DR+/38+/11b-/14-	12080	42	Low
7	72	NEG	NEG	NEG	NEG	NE	CD34+/33+/117+/14-	32000	53	
8	98	POS	NEG	NEG	NEG	46,XX,del(12)(p11p13)[13]/46,XX[7]	CD13+/33+/117+/DR4+/38+	3750	54	High
9	73	NEG	NEG	NEG	NEG	47,XY,t(11;19)(q23;p13),+der(19)t(11;19)(q23;p13) [21] 46,XY [3]	CD64+/13+/56+/11b+/33+/117+/−14±/DR+/4+/38+	1170	18	High
10	90%	NEG	NEG	NEG	POS (A)	46,XY,inv(16)(p13q22)[17]/47, inv(16)(p13q22),+mar[3]	64±/34+/13+/117±/14±/DR+/4+/38+/2+	88000	60	Low
11		POS	NEG	NEG	NEG	46,XX[20]	NE	122000	50	High

NE: not evaluable; ELN-RISK: European Leukemia Net risk category.

### Oxidative stress in AML CD34+ and normal CD34+ cells upon treatment with ASC+/−ATO

**Figure 3:**
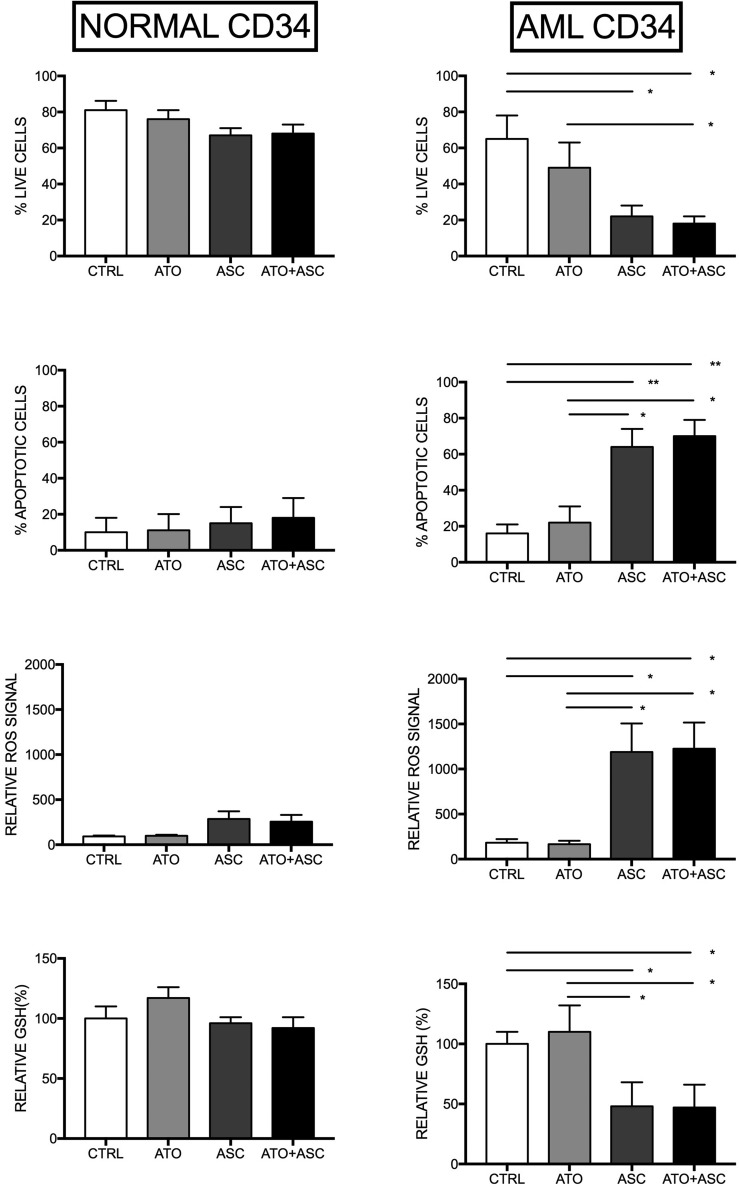
Selective oxidative stress induction in leukemic CD34^+^ cells Purified normal human CB CD34^+^ cells (left panels) and 5 AML CD34^+^ (right panel) were treated with no additives, 1 mM ATO or ATO+ASC at the indicated concentrations and analyzed after 24 h of incubation for the evaluation of the number of live cells, the percentage of apoptotic cells and GSH content, or immediately after drug addition for evaluation of ROS production. The percentage of apoptotic cells is obtained by flow cytometry analysis of Annexin V-labelled cells, while the percentage of viable cells is obtained by microscopy analysis of cells after staining with Trypan blue; ROS production was measured over a 2 hr time-lapse and peak values are plotted. The results reported in the figure represent the mean values ± SD observed in four separate experiments. *p < 0.05 **p < 0.01 ***p < 0.001 by the Anova *t* test and Tukey's Multiple Comparison Test.

In order to further explore the effects of ASC, alone and in combination with ATO, we focused on their effects in CD34^+^ AML blast cells and their normal counterpart, i.e. hematopoietic progenitors derived from human cord blood. Treatment with 1 μM ATO did not change the oxidative balance of the cells, whereas 3 mM ASC, alone or in combination with 1 μM ATO, resulted in an immediate outburst of ROS in CD34^+^ cells from AML specimens. In contrast, treatment of CD34^+^ cells derived from normal cord blood with 3 mM ASC, alone or in combination with ATO, induced only weak ROS production, resulting in a slight decrease of cell viability and induction of apoptosis, indicating a significant stronger oxidative challenge of the ASC/ATO combination on leukemic stem cells, compared to normal stem cells (Figure 3). As a control, we also used normal CD34+ cells isolated from healthy bone marrow (BM), confirming that these cells display low sensitivity to ASC-mediated oxidative stress, very similar to what observed using cord blood CD34+ cells (Supplementary Figure 1). In some experiments, the addition of the antioxidant N-Acetyl-Cysteine (NAC) to ASC, blocked ASC-mediated ROS production and induction of cell death (data not shown).

### ROS production and baseline myeloperoxidase levels in APL and AML

**Figure 4:**
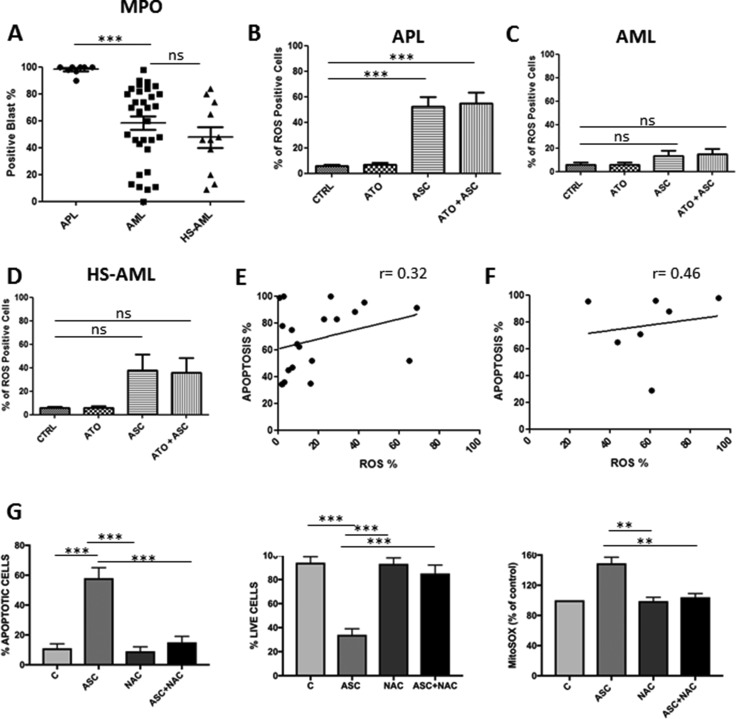
Correlation between apoptosis induced by ASC/ATO and levels of MPO or ROS production Basal levels of MPO in APL, AML and HS-AML blasts (**A**); % of ROS positive cells among primary APL blast population (**B**); % of ROS positive cells among primary AML blast population (**C**); % of ROS positive cells among primary AML-HS blast population (**D**); correlation between apoptosis and ROS positive cells in primary APL blasts (**E**), correlation between apoptosis and ROS positive cells in primary AML blasts (**F**). NAC protects NB4 cells from ASC-mediated ROS production and induction of cell death. NB4 cells were incubated in the absence (C) or in the presence of either 3 mM ASC, 2 mM NAC or both and evaluated for mitochondria ROS production (MitoSOX labeling), and for viability and apoptosis at 24 hours (**G**). **p ≤ 0.001 ***p ≤ 0.0001 by.

After immunohistochemical staining, 98.1 ± 3.5 % of APL blasts resulted MPO-positive at baseline, compared to 58 ± 28.2% of AML and 47.6 ± 26.3% of HS-AML samples. We hypothesized that the increased peroxidase activity in APL blasts (Figure 4A) would potentiate the apoptotic effect by inducing ROS overproduction (Figures 4B, 4C and 3D). Indeed, after ASC treatment, the median percentage of ROS-positive cells increased to 52 ± 21% in APL and 18 ± 22% in AML samples (*p* = 0.004). Consistently, combined treatment with ASC/ATO was associated to 54.7 ± 25% ROS-positive cells in APL and 19 ± 21% in AML samples (*p* = 0.003). However, the level of MPO or ROS production did not predict sensitivity to ASC in AML samples (Figure 4A, 4B, 4C and 4D) as the percentage of ROS-positive cells did not correlate with the percentage of apoptotic cells by ASC (Spearman's correlation 0.32; *p* = 0.17, Figure 4E). Furthermore, we found no significant correlation between the percentage of ROS-positive cells and the percentage of apoptotic cells in APL samples (Spearman's correlation 0.46; *p* = 0.30) (Figure 4F). To confirm that ASC-mediated ROS production was responsible for ASC-induced cell death, we used again the anti-oxidant NAC. The addition of 2 mM NAC to NB4 cells media prevented ASC-mediated ROS production assessed by MitoSOX labeling and induction of cell death (Figure 4G). This provided direct evidence that ASC mediated ROS production was responsible for ASC induced cell death.

### ROS production and GSH content in normal and leukemic cells after ASC/ATO

**Figure 5:**
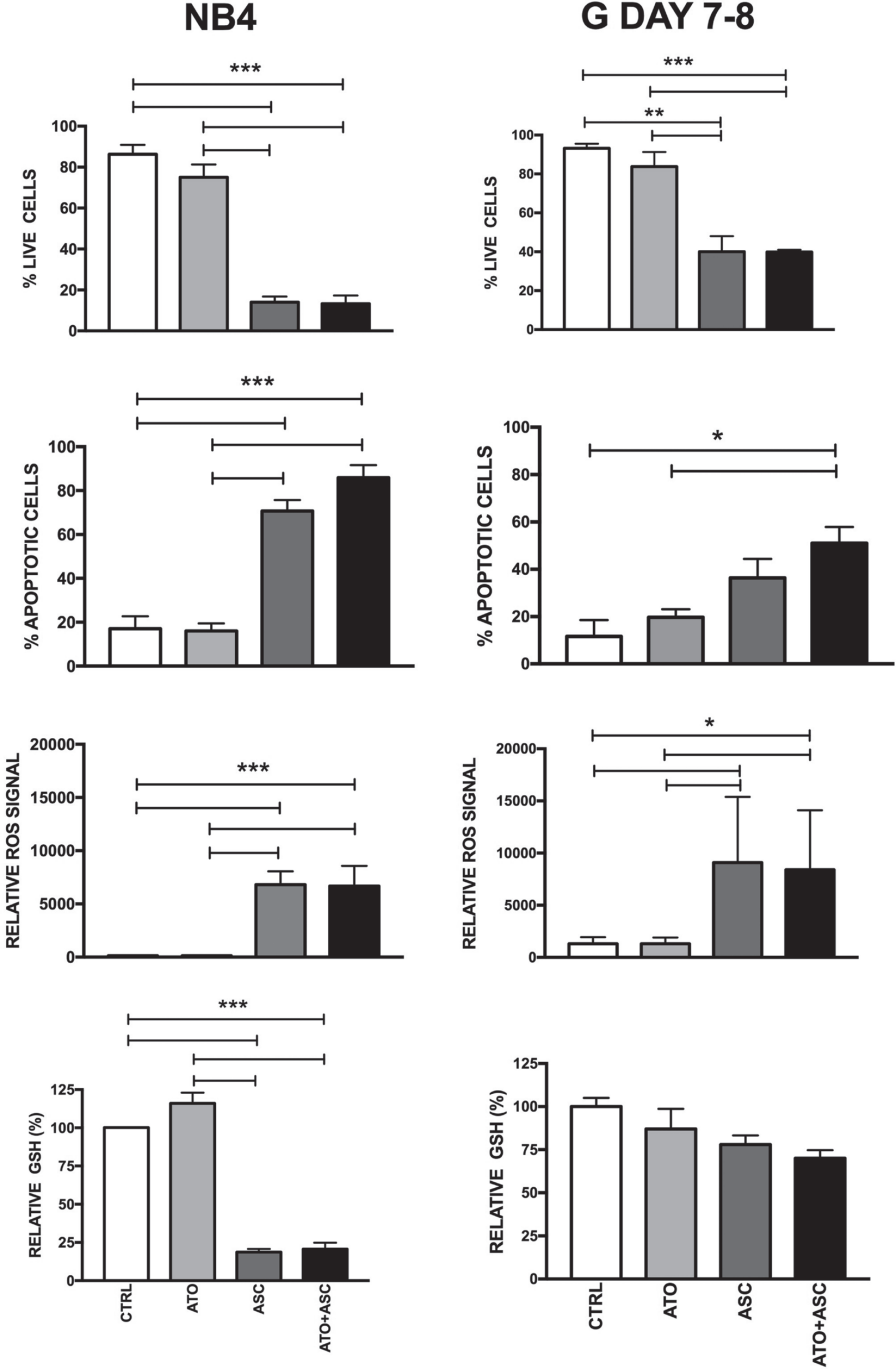
Sensitivity of normal and leukemic (NB4 cells) promyelocytes to ASC-mediated oxidative stress Normal CB CD34^+^ cells were grown for 7–8 days under unilineage granulocytic cell culture conditions. At that time, the majority of cells displayed cell morphology and immunophenotype typical of promyelocytes. The cells were then incubated and analyzed as indicated in the legend to Figure 3 (right panels). For comparison, the sensitivity of NB4 cells to ASC-mediated oxidative stress is reported (left panels). *p < 0.05 **p < 0.01 ***p < 0.001 by the Anova *t* test and Tukey's Multiple Comparison Test.

Given the higher sensitivity of APL to ASC and ATO/ASC, as compared to non-APL AML, we evaluated whether this may reflect higher “physiological” sensitivity of normal promyelocytes to these agents. To this end, we grew normal CB CD34^+^ cells under cell culture conditions that allow selective generation of granulocytic elements and, when the majority of differentiating granulocytic precursors reached the promyelocytic stage of differentiation, we evaluated their sensitivity to ASC and ATO exposure. Morphologic and immunophenotypic characterization of cells grown under these conditions has been previously described in detail by our group [33]. ASC elicited markedly higher ROS production in normal promyelocytes compared to CD34^+^ cells, associated with significant induction of apoptosis (Figure 5). While ASC and ATO/ASC were unable to induce a significant decrease of cellular GSH content in normal CD34+ cells, a moderate decrease of GSH was induced by these drugs in normal promyelocytes (Figure 5). When comparing to the APL cell line NB4, ROS activation by ASC and ATO/ASC was similar to that observed in normal promyelocytes, but GSH depletion and apoptosis were more pronounced than in normal promyelocytes (Figure 5). These results clearly show that leukemic promyelocytes are more sensitive than normal promyelocytes to ASC and ATO/ASC treatments.

### Analysis of Nrf2, SOD1 and catalase levels and response to ASC/ATO

We next tested the basal levels of the nuclear factor (erythroid-derived 2)-like 2 (Nrf2), a transcription factor that mediates ROS-induced cellular response, and of the ROS scavenger enzymes super oxide dismutase 1 (SOD1) and catalase in leukemic blast cells. There was no significant correlation between sensitivity to ASC and Nrf2 mRNA level (mRNA in AML 0.9 ± 0.7; HS-AML 1.3 ± 0.9; APL 1.1 ± 0.8) (Supplementary Figure 2A). On the other hand, Nrf2 protein levels were not significantly different in APL, compared to other AML subtypes likely due to the small sample size (Supplementary Figure 2B). SOD1 mRNA levels were also similar in the different samples (Supplementary Figure 2C). Catalase protein levels showed a trend towards lower levels in APL and HS-AML (Supplementary Figure 2D). Together, these data suggest that the pro-apoptotic effect of ASC/ATO treatment involves more complex mechanisms than simple ROS accumulation, that are largely dependent upon the intrinsic sensitivity of leukemic cells to oxidative stress.

### ASC treatment causes inhibition of activated FLT3 signaling

**Figure 6:**
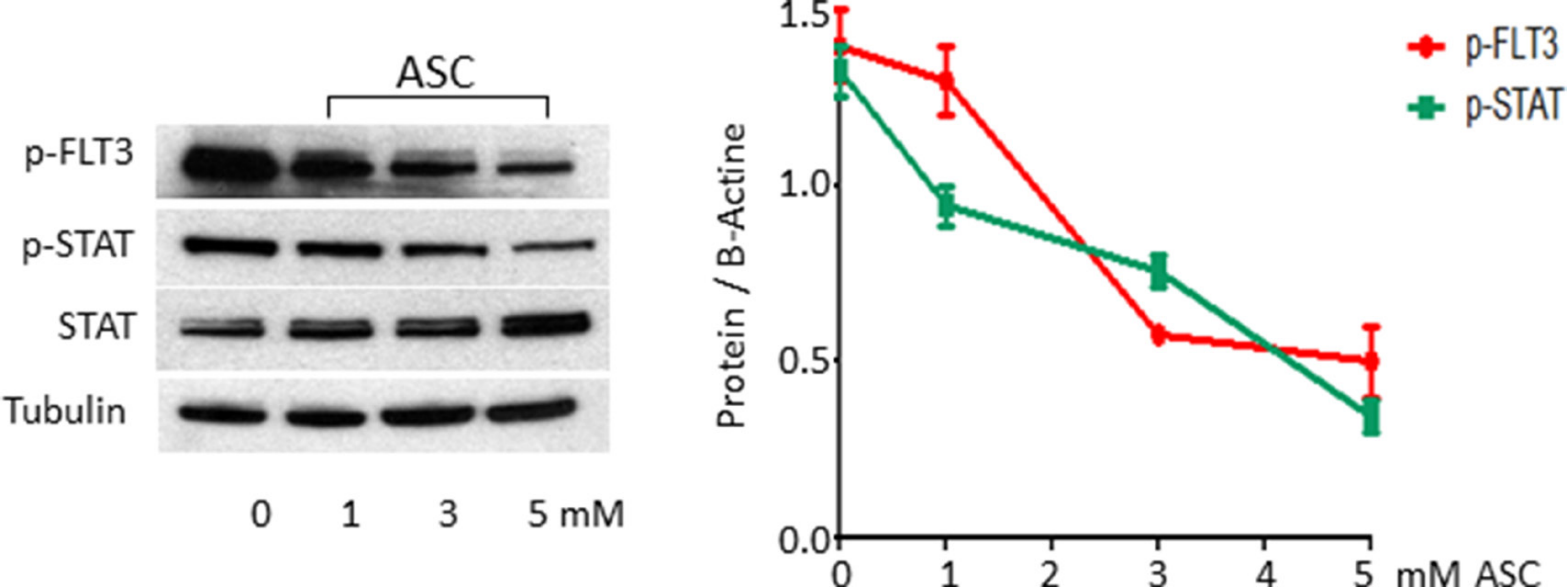
ASC treatment causes inhibition of activated FLT3 signaling The FLT3-ITD mutated MV4;11 cell line was treated for 24 hours with increasing ASC doses, ranging from 0.2 μmol/L up to 5 μmol/L, and then analyzed by Western blot analysis, using FLT3, p-FLT3, STAT5 and p-STAT5 antibodies.

Since high dose ASC affects growth in leukemic cells with constitutive activation of FLT3 signaling (MV4:11 cells and blasts from two primary samples FLT3-ITD positive), we treated MV4;11 cells with ASC at different concentrations. Among mechanisms activated by FLT3-ITD, we analyzed the STAT5a/b pathway, whose phosphorylation induces expression of target genes such as cyclin D1, c-myc and the anti-apoptotic gene p21, which are important for cell growth [34, 35]. Treatment with 3 mM and 5 mM ASC downregulated the FLT3 protein level and STAT5a-b phosphorilation (Figure 6).

### ASC treatment causes degradation of the PML/RARA hybrid protein, which is prevented by caspase inhibition

**Figure 7:**
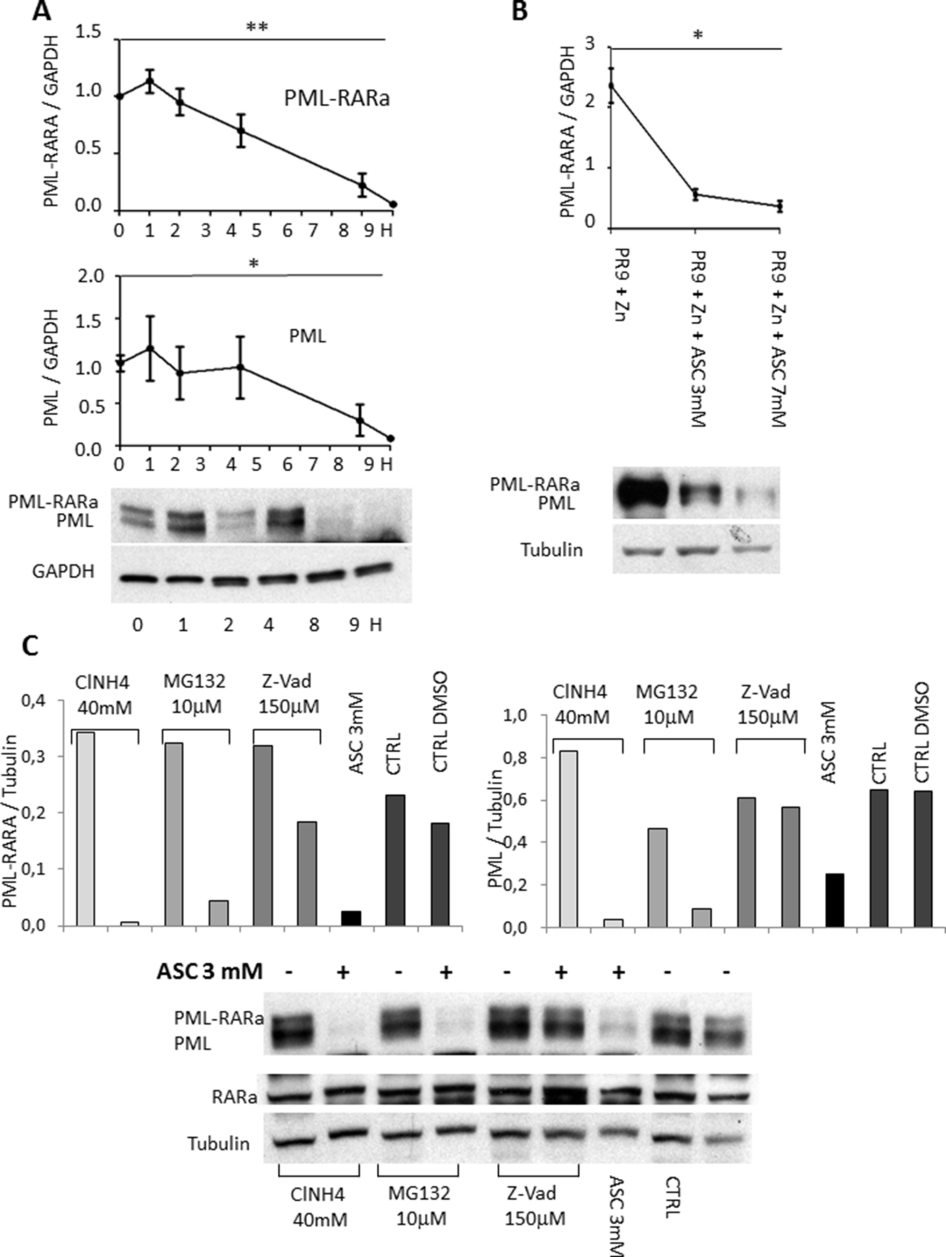

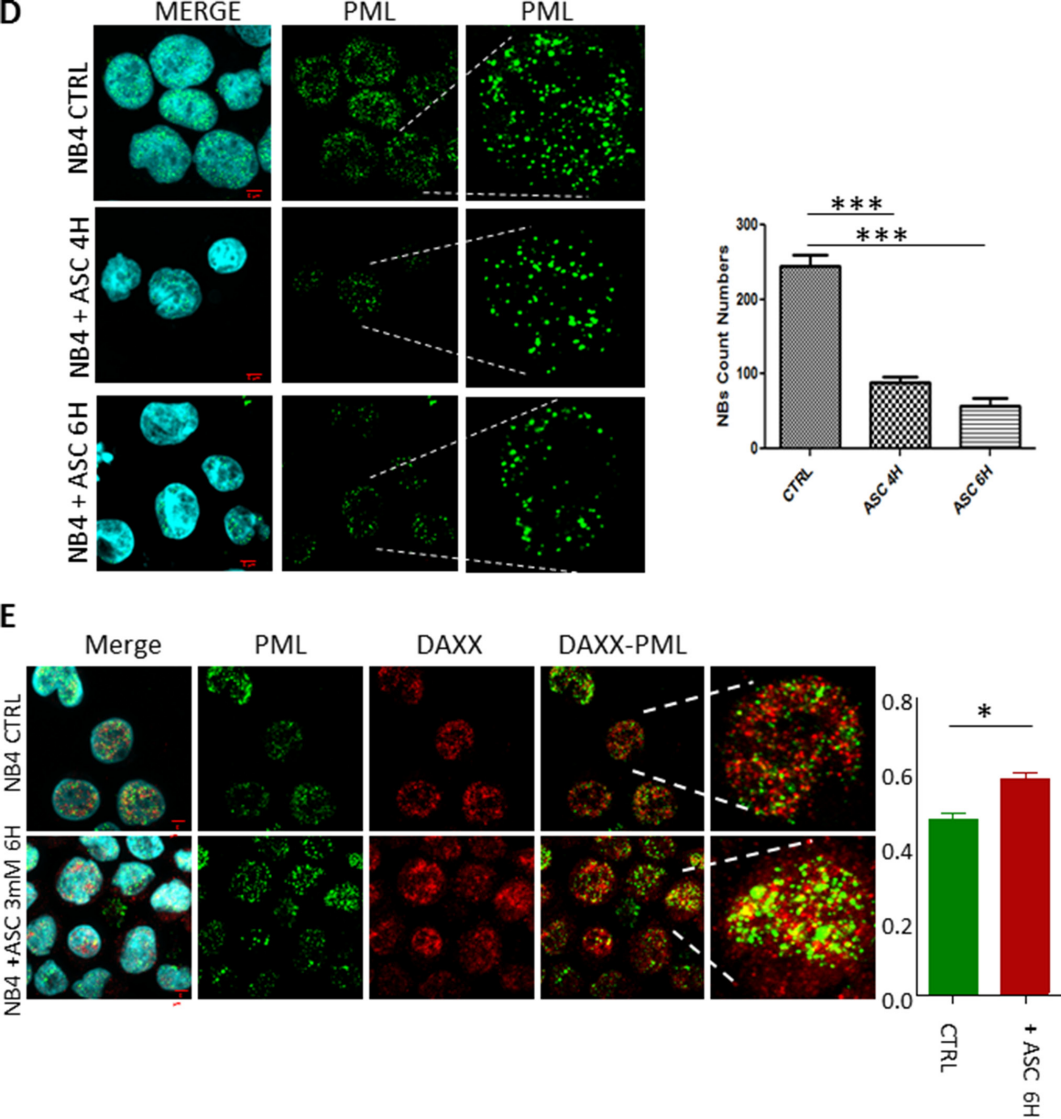
Degradation of PML/RARA hybrid and PML proteins following treatment with ASC NB4 cells exposed to 3 mM ASC: after 8 h PML/RARA is degraded by 80%, and PML by 70% (**A**). In PR9 cells treated with ZnSO_4_ for 2 h, PML/RARA is degraded after 2 h of treatment with 3 and 7 mM ASC (**B**). In NB4 cells treated with 3 mM ASC the proteasome inhibitor MG132, the autophagy inhibitor ClN4 and the caspase inhibitor Z-Vad were added. Only Z-Vad prevented ASC-induced degradation of PML and PML/RARA proteins *in vitro* (**C**). Using confocal microscopy, we observed reconstitution of PML NBs in NB4 cells after treatment with 3 mM ASC (**D**), and relocalization of DAXX in PML NBs (**E**). Colocalization of PML and DAXX was *p ≤ 0.05; ** ≤ 0.005 by unpaired *t* test. Quantification of number of nuclear bodies and colocalization, expressed in terms of overlap coefficient (R) was calculated on 30 randomly selected cells from different slides by using the WCIF ImageJ software (www.uhnresearch.ca/facilities/wcif/imagej/).

Since ASC treatment was particularly effective in APL samples, we investigated whether it interferes with the disease-unique PML/RARA oncoprotein. In NB4 cells challenged with 3 mM ASC, after an immediate induction at 1 h, the PML/RARA protein levels gradually decreased, reaching 70% degradation at 8 h (*p* = 0.0002), associated to 70% reduction of the native PML levels (*p* = 0.001) (Figure 7A and Supplementary Figure 3). Using the PR9 inducible system, the PML/RARA protein was induced by 1 μM ZnSO_4_ and degraded by 78% after 2 h and 3 mM ASC (*p* = 0.04) (Figure 7B). Analyzing the blots we noticed as well a clear reduction of PML protein (Figure 7A and Figure 7B) To investigate the mechanisms of PML and PML/RARA protein degradation in NB4 cells treated with ASC, we added to the culture medium the proteasome inhibitor MG132, the autophagy inhibitor ClN4 and the caspase inhibitor Z-Vad. Only addition of 100 μmol/L of the Z-Vad selective caspase inhibitor prevented the ASC-induced degradation of PML and PML/RARA proteins *in vitro*. Densitometric analysis of Western blots using anti-PML and anti-RARA antibodies showed about 90% inhibition of ASC-induced PML degradation and 58% inhibition of PML/RARA degradation in NB4 cells by Z-Vad (Figure 7C). The putative effect of ASC on PML in OCI-AML2 cells was studied and degradation of PML was confirmed. We did not find any significant change in NBs number and general aspect in that PML/RARa free environment (Supplementary Figure 4) indicating that residual PML protein function allows the partial NBs reconstitution in PML/RARa bearing cells. Apparently even a small quantity of PML can recruit and assemble NBs apparatus.

### ASC induces reconstitution of nuclear bodies and DAXX recruitment

PML nuclear bodies (NB) disruption by the PML/RARA protein induces delocalization of the repressor of transcription DAXX, along with PML [36]. Since PML is essential for the proper localization of DAXX in the NB and its pro-apoptotic action, we investigated the effects of ASC on PML NB formation and DAXX localization. Using confocal microscopy, we observed partial reconstitution of the PML NBs (Figure 7D) and DAXX recruitment (Figure 7E) in NB4 cells treated with 3 mM ASC.

## DISCUSSION

We investigated the cytotoxic effects of high-dose ascorbate (ASC) with or without ATO in human AML and APL cells. Since ATO functions as a pro-oxidant factor downregulating ROS scavenging proteins and disrupting redox pathways [31, 37, 38], we reasoned that it could synergistically act in association with ASC. We treated AML cell lines and primary AML blasts using this combination. In addition, we postulated that ATO could be a good partner of ASC in APL due to its impairing effects on the cellular response to oxidative stress, shown by PML degradation. PML acts as stress sensor in normal cells facilitating mitochondrial complex II action on ROS production, and minimizing ROS accumulation and preserving the Keap1-Nrf2 cytoplasmic complex [39].

Blasts from APL patients were highly sensitive to the ASC/ATO combination. Similarly, this treatment was effective in about one third of AML samples. Unfortunately, we were not able to identify predictive features for improved sensitivity to ASC in our AML series. In fact, no correlation was found between the amount of ROS, Nrf2, catalase and SOD-1 enzymes, and apoptotic response in individual patient blasts. In MV4;11 cells, harboring FLT3-ITD mutation, ASC at high concentrations downregulated FLT3 phosphorylation and that of its p-Stat5a/b target proteins, indicating potential activity of the drug in the subset of FLT3-ITD positive AML, notoriously characterized by poor prognostic outcome.

ASC caused an immediate selective ROS outburst in leukemic stem cells, but not in normal CD34^+^ cord blood cells, which possess highly effective scavenging systems against ROS [40]. The observation that ASC and ASC/ATO induced only modest effects on viability and apoptosis of normal hematopoietic progenitors appears relevant, especially in light of their potential therapeutic implications. Our results are in keeping with the notion that both agents have favorable safety profiles in humans and do not induce significant myelosuppression. However, while low-dose ascorbate coupled to ATO has been used in pilot studies to treat AML [41] and multiple myeloma [42, 43], to the best of our knowledge no clinical studies combining ATO and high-dose ASC have been carried out in hematologic malignancies. Therefore, the presumed safety of the combination remains to be established in phase I studies. As to ASC used as single agent, several studies in solid tumors and lymphoma have shown that plasma concentrations in the mM range are well tolerated in humans and carry only minimal toxicity [23–27, 44].

Various recent studies have shown substantial abnormalities of oxidative metabolism in AML cells. Lagadinou and coworkers showed that spontaneous ROS production in AML is heterogeneous, with cells characterized by low ROS levels displaying properties of leukemic stem cells, indicating a condition of ‘dormant’ metabolism with dependency on mitochondrial respiration, rather than glycolysis to sustain their energetic metabolism and survival [45]. Hole and coworkers reported an increased superoxide production by AML blasts, compared to normal CD34^+^ cells, not generated by mitochondria, but by increased NADPH oxidase activity, associated with reduced GSH content [46]. Skrtic and coworkers analyzed the mitochondrial function in primary AML cells, showing remarkable differences between normal HSCs/HPCs and their leukemic counterpart: AML cells, including leukemic stem cells, had a higher mitochondrial mass and higher oxygen consumption compared to normal HPCs [47]. Importantly, AML cells have low spare capacity in their respiratory chain complexes and are more sensitive to oxidative stress induced by the fatty acid palmitate [40]. Our observations further support the view that AML cells, including the leukemic stem cell subpopulation, are more vulnerable than their normal counterpart to cell death induced by oxidative stress.

We found here that ASC combined to ATO had synergistic effect in the OCI-AML2 AML cell line. In the NB4 APL cell line, even though ASC was highly effective, the addition of ATO only slightly potentiated the effect of ASC, indicating a modest additive effect, as shown also in primary APL samples. The reason why ASC is so efficient in APL, while the combination with ATO does not lead to significant enhancement of the cytotoxic effect of ASC probably lies in the degrading action of ASC not only on the PML/RARA protein itself but also on native PML, with the subsequent partial overlapping effect of the two chemicals.

On the other hand, after ASC treatment the residual PML protein may partially recruit Nuclear Bodies integrants in NB4 cells, as shown by the partial reversion of the APL phenotype with reassembly of NBs and restoration of the PML/Death-associated protein 6 (DAXX) interaction. DAXX acts as a transcription regulator [48, 49] and interacts with chromatin remodeling pathways [HDAC-II, acetyltransferases (CBP), and DNA methyltransferase (Dnmt1) [50]. Thus, upon ASC treatment, DAXX relocalizes in the nuclear bodies and could favor apoptosis via an alternative mechanism on cell metabolism, aside from ROS induction.

In conclusion, our findings may provide a rationale to test a reportedly harmless pro-oxidant therapy, as ASC alone or in combination with ATO, in advanced myeloid malignancies including elderly AML, and APL resistant to conventional therapy.

## MATERIALS AND METHODS

### Cell lines and primary leukemia samples

NB4, an APL-derived cell line, carrying the t(15;17) translocation, and MV4;11, an AML-M5 derived cell line carrying the t(4;11) translocation and FLT3-ITD mutations were purchased from DSMZ (Braunschweig, Germany). The ATRA-resistant cell line, NB4-R4, was kindly donated by Dr. Clara Nervi (Sapienza University, Rome, Italy), while the ATO-resistant cell line, NB4-ATO-R, was established by culturing in increasing concentrations of ATO, with initial concentration of 200 nM, until the cell line survived a concentration of 1 μM. The resistant cell line was then maintained in complete RPMI 1640 medium containing 1 μM ATO. Oci-AML2, an AML-M4 derived cell line carrying the DNMT3A R635W mutation, was kindly provided by Dr. Emanuela Colombo (European Institute of Oncology, Milan, Italy).

All cells were grown at 37°C in a humidified atmosphere of 5% CO_2_ in air, in RPMI medium (GIBCO-BRL, Grand Island, NY, USA), supplemented with 10% fetal bovine serum (FBS) (GIBCO-BRL) 20 mM Hepes, 100 U/mL penicillin and 100 μg/mL streptomycin (GIBCO-BRL).

Bone marrow (BM) samples were collected from 48 consecutive patients diagnosed with *de novo* AML (*n* = 37), APL (*n* = 9) or CML (*n* = 2, 1 chronic phase and 1 blast crisis) admitted at the Department of Hematology of the University of Rome Tor Vergata. All samples had at least 70% infiltration by leukemic blasts. Written informed consent was obtained from all patients in accordance with the Declaration of Helsinki and the study was approved by the ethical committee of the University of Rome Tor Vergata.

### Genetic characterization of leukemic samples

Conventional karyotyping was performed and reported according to the International System for Human Cytogenetic Nomenclature [51]. For molecular diagnostic studies, total RNA was extracted from BM mononuclear cells separated by Ficoll–Hypaque, using the method of Chomczynsky and Sacchi [52]. DNA was extracted using a column-based kit (QIAmp DNA, Quiagen, Hilden, Germany). Samples were characterized for the presence of *BCR/ABL*, *PML/RARA*, *CBFB/MHY11*, *RUNX1/RUNX1T1*, and *DEK/CAN* fusion genes, and for *NPM1* and *FLT3*-internal tandem duplication (ITD) mutations using methods reported elsewhere [53–54].

### Immunophenotypic analysis

Immunophenotypic studies were performed on diagnostic bone marrow samples. Data were analyzed on a 9-Color CyAn ADP, equipped with three lasers (Beckman Coulter, Brea, CA). Briefly, samples were lysed with ammonium chloride (PH 7.2) and stained with pre-defined optimal concentrations of antibodies and dead cells stain dyes (LIVE/DEAD Fixable Aqua Dead Cell Stain Kit, Life Technologies, Eugene, USA). Each antibody was incubated with 0.5 × 10^6^ cells in a 50-μL volume, for 20–30 min. After 2 washings in phosphate-buffered saline (PBS), cells were re-suspended in 0.2 mL PBS and analyzed with the flow cytometer. The antibodies used are listed in Supplementary Table 1.

### Human progenitor cell (HPC) purification

Cord blood (CB) was obtained after informed consent from healthy full-term placentas according to institutional guidelines. BM-mononuclear cells were obtained from healthy donors. CD34^+^ cells were purified from CB or BM by positive selection using the midi-MACS immunomagnetic separation system (Miltenyi Biotec, Bergisch Gladabach, Germany) according to the manufacturer's instructions. The purity of CD34^+^ cells was assessed by flow cytometry using a phycoerythrin (PE)-conjugated anti-CD34 monoclonal antibody and was routinely over 95% (range comprised between 92–98%). Purified human hematopoietic progenitor cells were grown in serum-free medium containing BSA (10 mg/ml), pure human transferrin (1 mg/ml), human low-density lipoproteins (40 μg/ml), insulin (10 μg/ml), sodium pyruvate (10^−4^M), L-glutamine (2 × 10^−3^ M), rare inorganic elements (Sn, Ni, Va, Mo and Mn) supplemented with iron sulphate (4 × 10^−8^M) and nucleosides (10 μg/ml each). HPCs were induced into specific granulopoietic differentiation with IL-3 (1 unit/ml), granulocyte/monocyte GM-CSF (0.1 ng/ml) and saturating amounts of G-CSF (500 units/ml). The differentiation stage of unilineage cultures was evaluated by May Grunwald-Giemsa staining (Sigma-Aldrich, St. Louis, Mo, USA) and cytological analysis[33]. Leukemic CD34^+^ cells were purified from primary AML bone marrow samples using the same procedure used for purification of normal CB CD34^+^ cells.

### Survival assay

Primary blasts were cultured at a concentration of 1 × 10^6^/ml, at 37°C, in a humidified atmosphere of 5% CO_2_ in air in RPMI 1640 (GIBCO-BRL), supplemented with 20% fetal bovine serum (FBS) (GIBCO-BRL), 20 mM Hepes, 100 U/mL penicillin and 100 mg/mL streptomycin (GIBCO-BRL). Sodium L-Ascorbate (ASC) (Sigma-Aldrich Co) was dissolved in water at 1 M concentration immediately before use. ATO (Sigma-Aldrich Co) was dissolved in PBS at 1 mM concentration. ASC and ATO were added at the beginning of each experiment and left in the culture medium during the entire period of incubation.

For studies in cell lines, cells were plated in 96-well plates at 5000 cells/well, exposed to vehicle, 1 μm ATO or 3 mM ASC, or ATO plus ASC combination, and cultured for 3 days. Cell survival was evaluated by the CellTiter 96^®^ AQueous One Solution Cell Proliferation Assay Kit, according to the manufacturer's instructions (Promega, Madison, USA).

### ROS assay and apoptosis analysis

To evaluate ROS levels, cells were incubated for 4 h with ATO, ASC or the combination of the two. The Abcam's ROS assay kit “ab113851” (Abcam, Cambridge, UK) uses the cell permeant reagent 2′,7′–dichlorofluorescein diacetate (DCFDA), a fluorogenic dye, that measures hydroxyl, peroxyl and other ROS activity within the cell. After diffusion into the cell, DCFDA is deacetylated by cellular esterases to a non-fluorescent compound, which is later oxidized by ROS into 2′, 7′ –dichlorofluorescein (DCF). The cells were then analyzed using a Beckman Coulter CyAn ADP (Chapel Hill, USA).

Five hundred thousand cells were incubated with the vehicle, 1 μm ATO, 3 mM ASC, or 1 μm ATO plus 3 mM ASC. The cells were then harvested 48 h later and stained with the Annexin V-FITC apoptosis detection kit, according to manufactured instructions (eBioscience Ds, Bender Med Systems GmbH, Vienna, Austria), and analyzed using a Beckman Coulter CyAn ADP (Chapel Hill, USA). Intracellular glutathione (GSH) content was evaluated by a flow cytometry method based on the use of the thiol green dye, using the procedure recommended by the supplier (ABCAM, Co, USA).

### Western blot

Cell pellets were re-suspended in lysis buffer containing 10 mM Tris–HCl (pH 7.4), 5 mM EDTA, 150 mM NaCl, 1% Triton X-100, 250 μM orthovanadate, 20 mM β-glycerophosphate and protease inhibitors (Sigma-Aldrich). Lysates were centrifuged at 10,000 g for 15 min at 4°C and supernatants were stored at −80°C. Thirty μg aliquots of proteins were resuspended in a reducing Laemmli Buffer and loaded onto a 10% polyacrylamide gel, then transferred to a nitrocellulose membrane. The membranes were incubated with specific antibodies [anti-Nrf2 (C-20): sc-722 Santa Cruz Biotechnology, Inc. Texas, USA; anti-catalase (Gene Tex, Inc., GTX110704, Texas, USA; anti-PML (H-238): sc-5621, Santa Cruz Biotechnology; anti Phospho Stat5a/b(R&D Systems Inc, Minneapolis, USA); anti STAT5a/b (R&D Systems Inc, Minneapolis, USA); anti p-FLT3 Tyr 591 (Cell Sygnaling Technology, Beverley, MA, USA); anti FLT3 Ligand (R&D Systems Inc, Minneapolis, USA)]. Horseradish peroxidase-conjugated IgG preparations were used as secondary antibodies and the enhanced chemiluminescence (ECL) procedure was employed for development (ECL kits, Amersham, Buckinghamshire, UK). The autoradiograms were scanned and exported for densitometry analysis. Protein signal intensities were measured using Quantity One Software (Bio-Rad Laboratories, Hercules, CA, USA). Signal quantity was normalized against the unrelated proteins GAPDH and α-tubulin (Abcam plc).

### Quantitative Real-Time PCR

Reverse transcription was performed using 1 μg total RNA and a standardized protocol (Applied Biosystems, Foster City, CA). The primers and probes were obtained from Integrated DNA Technology (IDT) (Supplementary Table 2). The reaction mixture of 20 μl contained 1 × Master Mix (Applied Biosystems), 300 nM of each primer, 200 nM of Taqman polymerase. All expression levels (gene of interest and normalization control) in quantitative RT–polymerase chain reactions (RQ-PCR) were obtained by the use of standard curves.

### PML and DAXX nuclear staining pattern

Cytospins were prepared from the NB4 cell line using a cytocentrifuge. Cells fixed with 4% paraformaldehyde (PFA) were permeabilized in PBS containing 0.2% Nonidet P-40 and blocked in 5% BSA. Slides were incubated overnight with the primary antibody [PG-M3 [55] anti-PML, kindly provided by Brunangelo Falini; and anti- PML/Death-associated protein 6 (DAXX, Cell Signaling Technology, Beverley, MA, USA)], washed twice in PBS and incubated for 2 h with secondary antibodies: Invitrogen Alexa Fluor 555-labeled goat anti-mouse and Invitrogen Alexa Fluor 488-labeled goat anti-rabbit. After nuclear counterstaining with 4′,6-diamidino-2-phenylindole (DAPI), slides were visualized using an Olympus BX61 fluorescent microscope equipped with a CoolSNAP EZ camera (Photometrics, Tucson, AZ, USA). Quantification of the number of nuclear bodies and colocalization, expressed in terms of overlap coefficient (R), was assessed on several randomly selected cells from different slides by using the WCIF ImageJ software (www.uhnresearch.ca/facilities/wcif/imagej/).

### Statistical analysis

The dose-effect curves of ASC or ATO single-agents and their combination, were analyzed by the median-effect method of Chou and Talalay [56] using the Calcusyn Software (Biosoft, Cambridge, UK). The combination index (CI) indicates a quantitative measure of the degree of drug interaction in terms of synergistic (CI < 1), additive (CI = 1) or antagonistic effect (CI > 1). The calculation of CI was performed using a non-constant ratio combination, testing graded concentrations of ASC with a fixed non toxic concentration of ATO. All other analyses were conducted using the GraphPad Prism Program.

## SUPPLEMENTARY MATERIALS FIGURES AND TABLES


