# Supplementary material for: High-dose ascorbate and arsenic trioxide selectively kill acute myeloid leukemia and acute promyelocytic leukemia blasts in vitro
Source: Oncotarget. 2017 Mar 6;8(20):32550–65. doi: 10.18632/oncotarget.15925 (PMC5464808; doi:10.18632/oncotarget.15925)
Supplement: Supplementary file 1 [file oncotarget-08-32550-s001.pdf]

High-dose ascorbate and arsenic trioxide selectively kill acute myeloid leukemia and acute promyelocytic leukemia blasts *in vitro*

Supplementary Materials

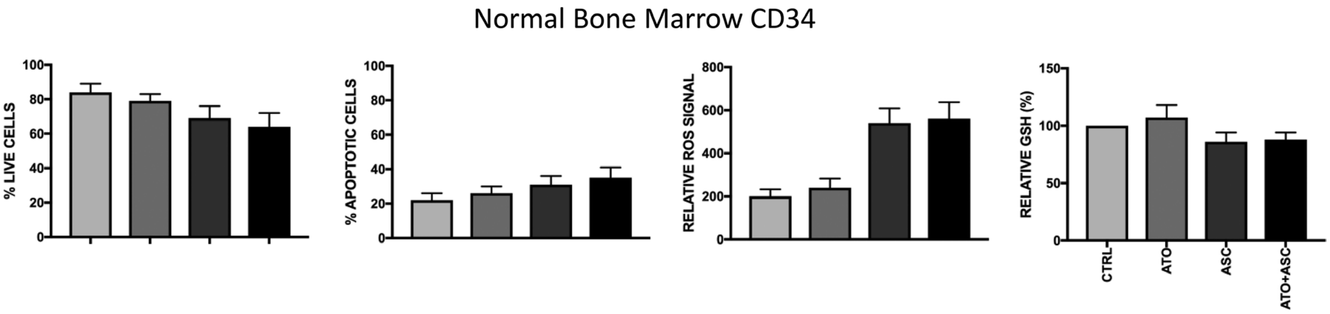

Supplementary Figure 1: Oxidative stress induction in Normal Bone Marrow CD34$^+$ cells. Purified Normal Bone Marrow (NBM) CD34$^+$ cells, were treated with no additives, 1 µM ATO or ATO+ASC at the indicated concentrations and analyzed after 24 h of incubation for the evaluation of the number of live cells, the percentage of apoptotic cells and GSH content, or immediately after drug addition for evaluation of ROS production. ROS production was measured over a 2 hr time-lapse and peak values are plotted.

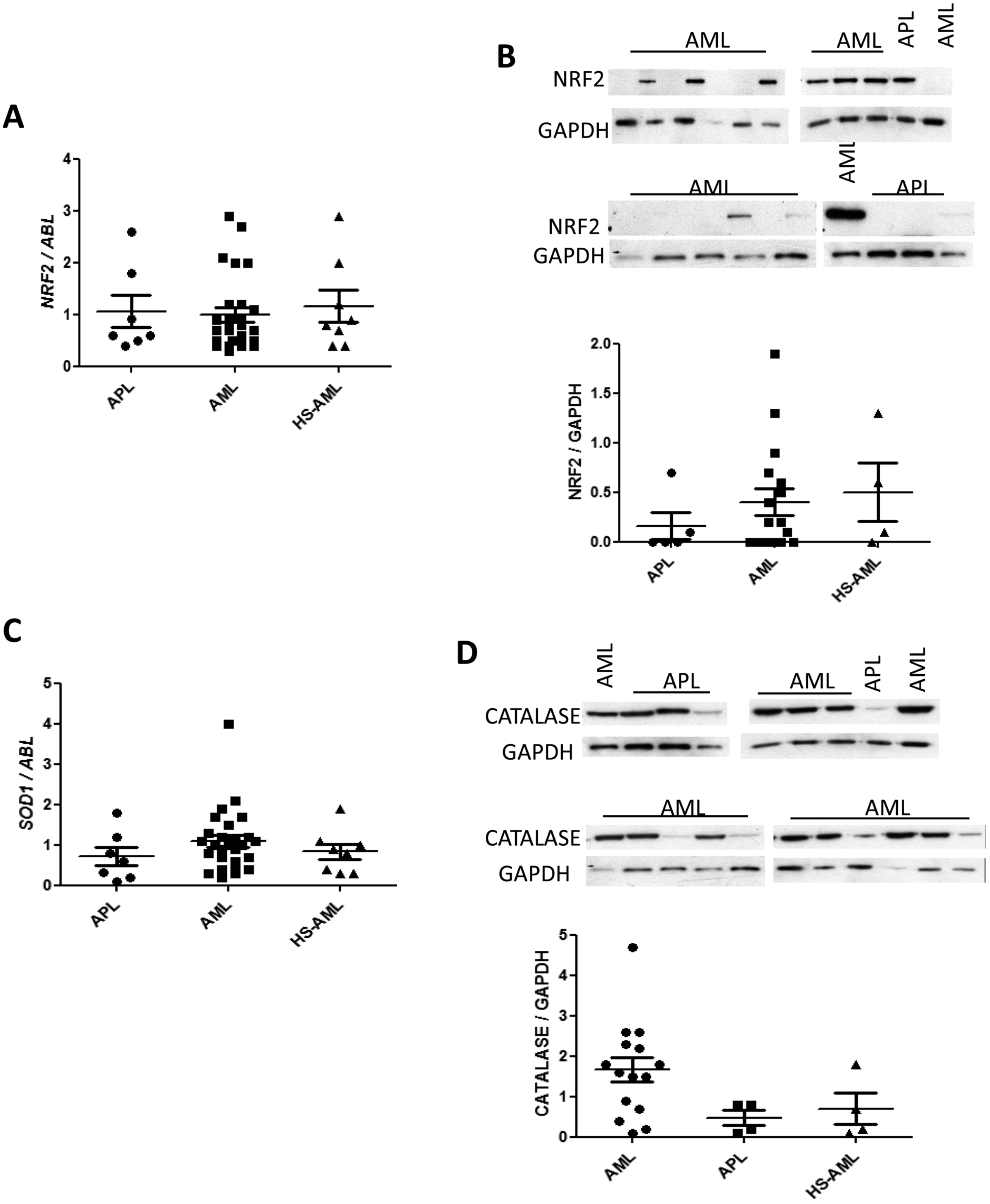

Supplementary Figure 2: ROS metabolism gene products in primary blasts. *Nrf2* mRNA expression in primary APL and AML blast cells (**A**); Nrf2 protein level in primary APL and AML blasts (**B**); *SOD1* mRNA expression in primary APL and AML blasts (**C**); catalase protein level in primary APL and AML blasts (**D**).

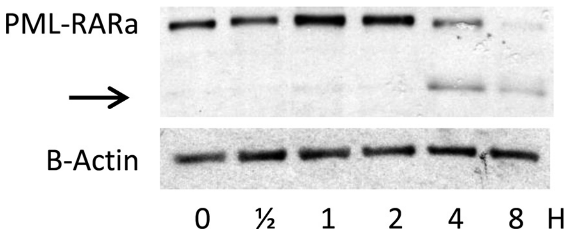

Supplementary Figure 3: PML/RARa degradation in NB4 cells after ASC treatment. NB4 cells were treated with ASC 3 mM and analyzed for PML/RARa expresssion by immunoblotting using the anti RARa antibody.

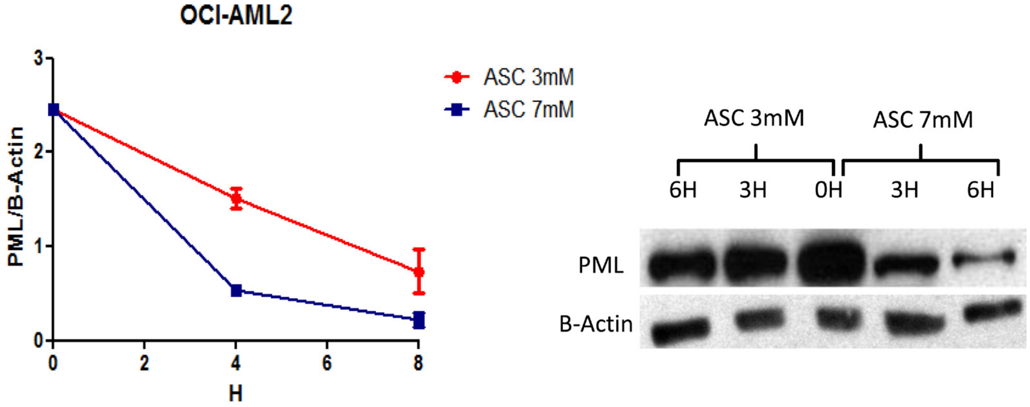

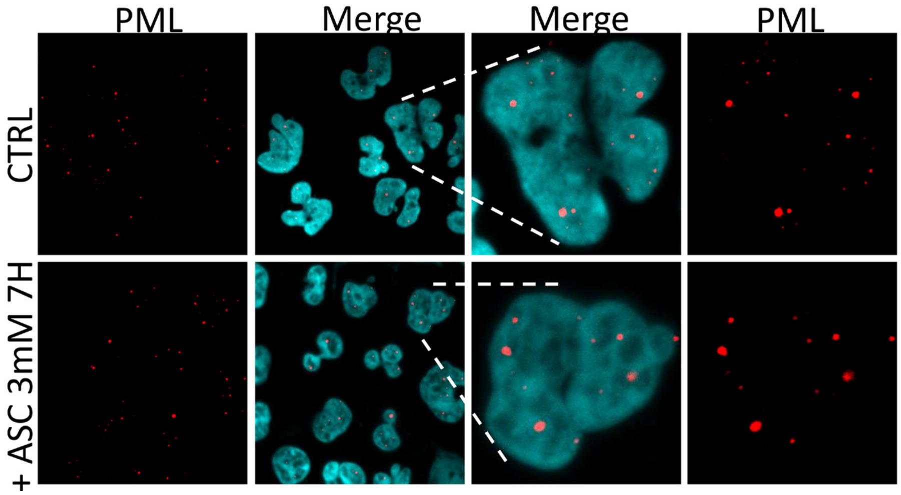

Supplementary Figure 4: Degradation of PML proteins in Oci-AML2 cells following treatment with ASC. OCI-AML2 cells exposed to 3 mM and 7 mM ASC PML is degradated (**A**). Using confocal microscopy in OCI-AML2 cells after treatment with 3 mM ASC, we doesn't observed significant difference compear to the control in PML NBs (**B**).

Supplementary Table1: Anti-human antibodies used

Antigen	Fluorochrome	Clone	Purchased from
CD45	CD45 APC-eFluor 780	HI30	e-Bioscience
CD33	PE	AC104.3e3	Miltenyi
CD38	PerCP/Cy5.5	HIT2	Biolegend
CD117(C-Kit)	PE-CY7	104D2	Beckman Coulter
HLADR	ECD	IMMU357	Beckman Coulter
CD34	APC	8G12	BD Pharmingen
CD11b	PE7Cy5.5	ICRF44	BioLegend
CD64	FITC	10.1	BD Pharmingen
CD4	Pacific Blue	13b8.2	Beckman Coulter
CD15	FITC	80H5	Beckman Coulter
CD13	PE/Cy5.5	SJ1D1	Beckman Coulter
CD14	PE-Cy7	RMO52	Beckman Coulter
CD7	PE	CBIO1241D1	e-Bioscience
CD2	FITC	RPA-2.10	e-Bioscience

Supplementary Table 2: Sequence of primers and probes

ABL-forward	5′- TGGAGATAACACTCTAAGCATAACTAAAGGT-3′
ABL-reverse	5′-GATGTAGTTGCTTGGGACCCA-3′
ABL-probe	5′-FAM- CCATTTTTGGTTTGGGCTTCACACCATT-BHQ-3
Nrf2-forward	5′-TGGAACAGAGAAAGCAGAGTG-3′
Nrf2-reverse	5′-TGTAGTCTTGGTTCTAGCAGTTTC-3′
Nrf2-probe	5′-FAM-TGGTTCATT/ZEN/GATGTCTATGGCCTGGC/3IABkFQ-3′
SOD1-forward	5′-CCTCGGAACCAGGACCT-3′
SOD1-reverse	5′-TTAATGCTTCCCCACACCTT-3′
SOD1-probe	5′-FAM/TCGCCATAA/ZEN/CTCGCTAGGCCAC/3IABkFQ-3′
